# Identification of five novel variants of *ADAR1* in dyschromatosis symmetrica hereditaria by next-generation sequencing

**DOI:** 10.3389/fped.2023.1161502

**Published:** 2023-07-05

**Authors:** Qian Ma, Lingyi Che, Yibing Chen, Zhuoyu Gu

**Affiliations:** ^1^ Genetic and Prenatal Diagnosis Center, Department of Gynecology and Obstetrics, The First Affiliated Hospital, Zhengzhou University, Zhengzhou, China; ^2^ Department of Thoracic Surgery, The First Affiliated Hospital of Zhengzhou University, Zhengzhou University, Zhengzhou, China

**Keywords:** next-generation sequencing, dyschromatosis ymmetrica hereditaria, *ADAR1*, mutations, hyperpigmented and hypopigmented

## Abstract

**Background:**

Dyschromatosis symmetrica hereditaria (DSH) is a rare autosomal dominant inherited pigmentary dermatosis characterized by a mixture of hyperpigmented and hypopigmented freckles on the dorsal aspect of the distal extremities. To date, pathogenic mutations causing DSH have been identified in the adenosine deaminase acting on RNA1 gene (*ADAR1*), which is mapped to chromosome 1q21.

**Objective:**

The present study aimed to investigate the underlying pathological mechanism in 14 patients with DSH from five unrelated Chinese families. Next-generation sequencing (NGS) and direct sequencing were performed on a proband with DSH to identify causative mutations. All coding, adjacent intronic, and 5′- and 3′-untranslated regions of *ADAR1* were screened, and variants were identified.

**Result:**

These mutations consisted of three missense mutations (NM_001025107: c.716G>A, NM_001111.5: c.3384G>C, and NM_001111.5: c.3385T>G), one nonsense mutation (NM_001111.5:c.511G>T), and one splice-site mutation (NM_001111.5: c.2080-1G>T) located in exon 2, exon 14, and the adjacent intronic region according to recommended Human Genome Variation Society (HGVS) nomenclature. Moreover, using polymerase chain reaction and Sanger sequencing, we identified five novel *ADAR1* variants, which can be predicted to be pathogenic by in silico genome sequence analysis. Among the mutations, the missense mutations had no significant effect on the spatial structure of the protein, while the stop codon introduced by the nonsense mutation truncated the protein.

**Conclusion:**

Our results highlighted that the advent of NGS has facilitated high-throughput screening for the identification of disease-causing mutations with high accuracy, stability, and specificity. Five novel genetic mutations were found in five unrelated families, thereby extending the pathogenic mutational spectrum of *ADAR1* in DSH and providing new insights into this complex genetic disorder.

## 1. Introduction

Dyschromatosis symmetrica hereditaria (DSH, OMIM#127400) or symmetric dyschromatosis of the extremities is a rare autosomal dominant skin disease with high penetrance (1). DSH was first discovered by Toyama in 1910 and formally named as a clinical entity in 1929; the disease is characterized by a mixture of hypopigmented and hyperpigmented macules on the dorsa of the hands and feet (2). Some patients may also have small pigmented macules on their face. Exposure to sunlight can even cover the entire body and exacerbate the lesion. Skin lesions normally appear in infancy or early childhood, aggravate in adolescence, and last for lifetime (3, 4). DSH has been reported worldwide, mostly in Japanese and Chinese populations (5–8). It’s reported that DSH is caused by *ADAR1* gene mutations from birth. It is thought to be associated with *ADAR1* haploinsufficiency, as well as pathogenic effects of mutation though the exact mechanism of this disease remains unknown. The epidermal symptoms can be easily overlooked in patients since it is influenced by confounding factors such as skin colour changes, duration, disease severity and progression.

Adenosine deaminase acting on RNA1 gene (*ADAR1*) encodes a double-stranded RNA-editing enzyme that catalyzes the conversion of adenine to hypoxanthine and is located on chromosome 1q21.3 (9). It has two different transcription initiation sites, forming two subtypes: P150 and P110 (10). Cumulative studies have shown that the P150 isoform plays a critical role in the development of DSH (11). Defective *ADAR1*-mediated editing leads to disorders such as Aicardi-Goutières syndrome (AGS), autoinflammatory diseases of the skin, and DSH, which manifests as a skin pigmentation disorder (12). Currently, *ADAR1* is expressed in different human cells, and mutations in this gene underlie the pathogenesis of DSH; however, the molecular mechanism underlying the pathogenesis this disease has not yet been completely elucidated (13–15).

Next-generation sequencing (NGS) technologies have been established to analyze the sequences of exons and intronic regions of genes as a primary testing modality in genomic prognosis with clinical relevance. The use of NGS leads to lower costs, higher turn-around efficiency, several more orders of magnitude, and more comprehensive coverage of large-scale sequencing than conventional techniques (16, 17). In this study, we carried out NGS to screen genes associated with DSH and identified five novel mutations in *ADAR1*. Our findings emphasize the importance of NGS, which can be a useful tool for the identification of pathogenic gene variants and genetic diagnosis. The results expanded the spectrum of known gene variants involved in DSH, and this could help investigate phenotype-genotype relationships and aid in genetic counselling of patients with DSH.

## 2. Materials and methods

### 2.1. Ethical compliance

The study and procedures conformed to the tenets of the Declaration of Helsinki and were approved by the Research Ethics Committee of the First Affiliated Hospital of Zhengzhou University (KS-2018-KY-36). All subjects in our study signed the informed consent for the publication of this article.

### 2.2. Subjects and clinical evaluation

**Figure 1:**
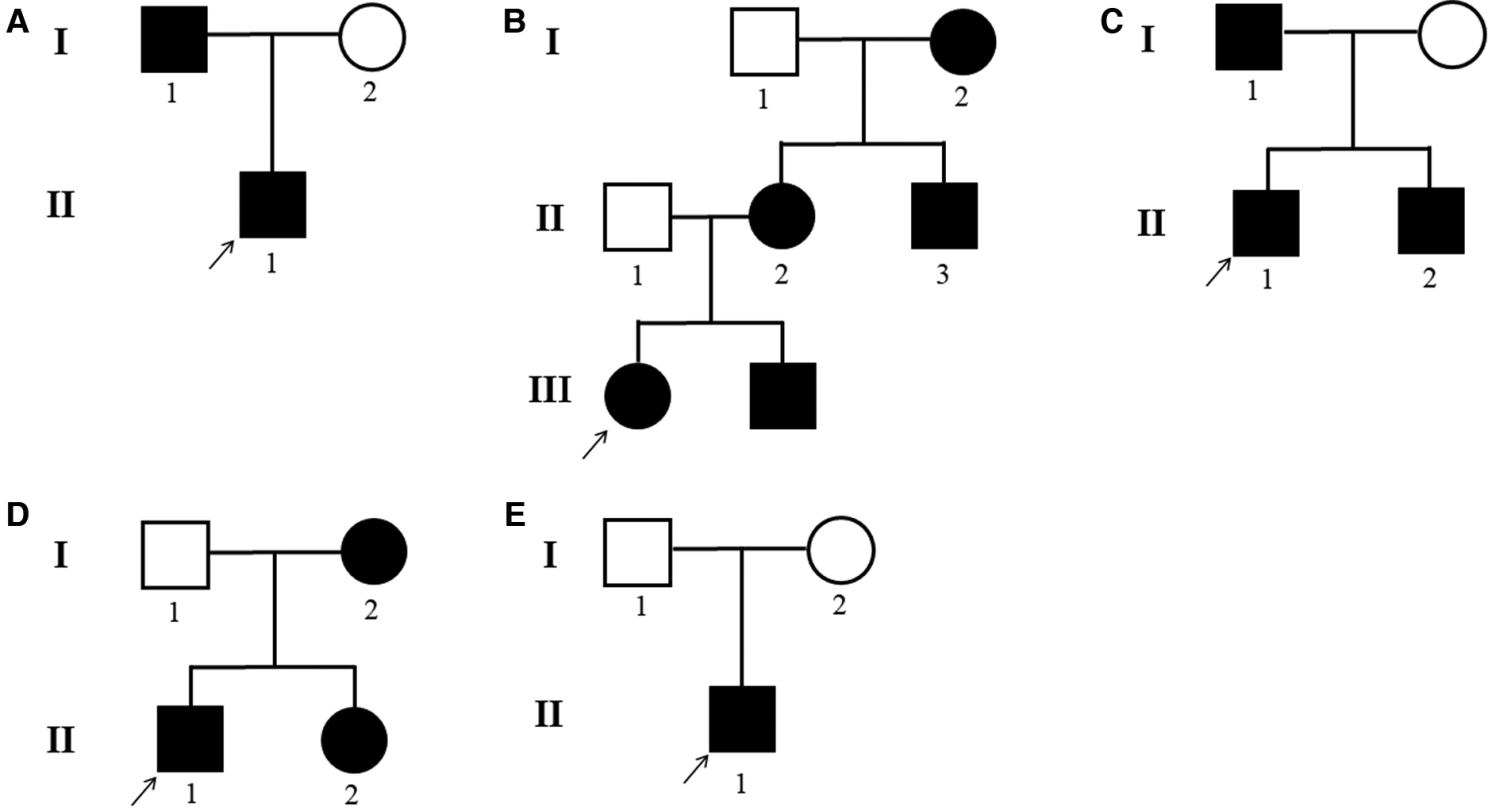
(**A**–**E**) DSH pedigrees of the five families. Black circle: affected mutation-carrying female; white circle: female without *ADAR1* mutation; Black square: affected mutation-carrying male; white square: male without *ADAR*1 mutation.

**Figure 2:**
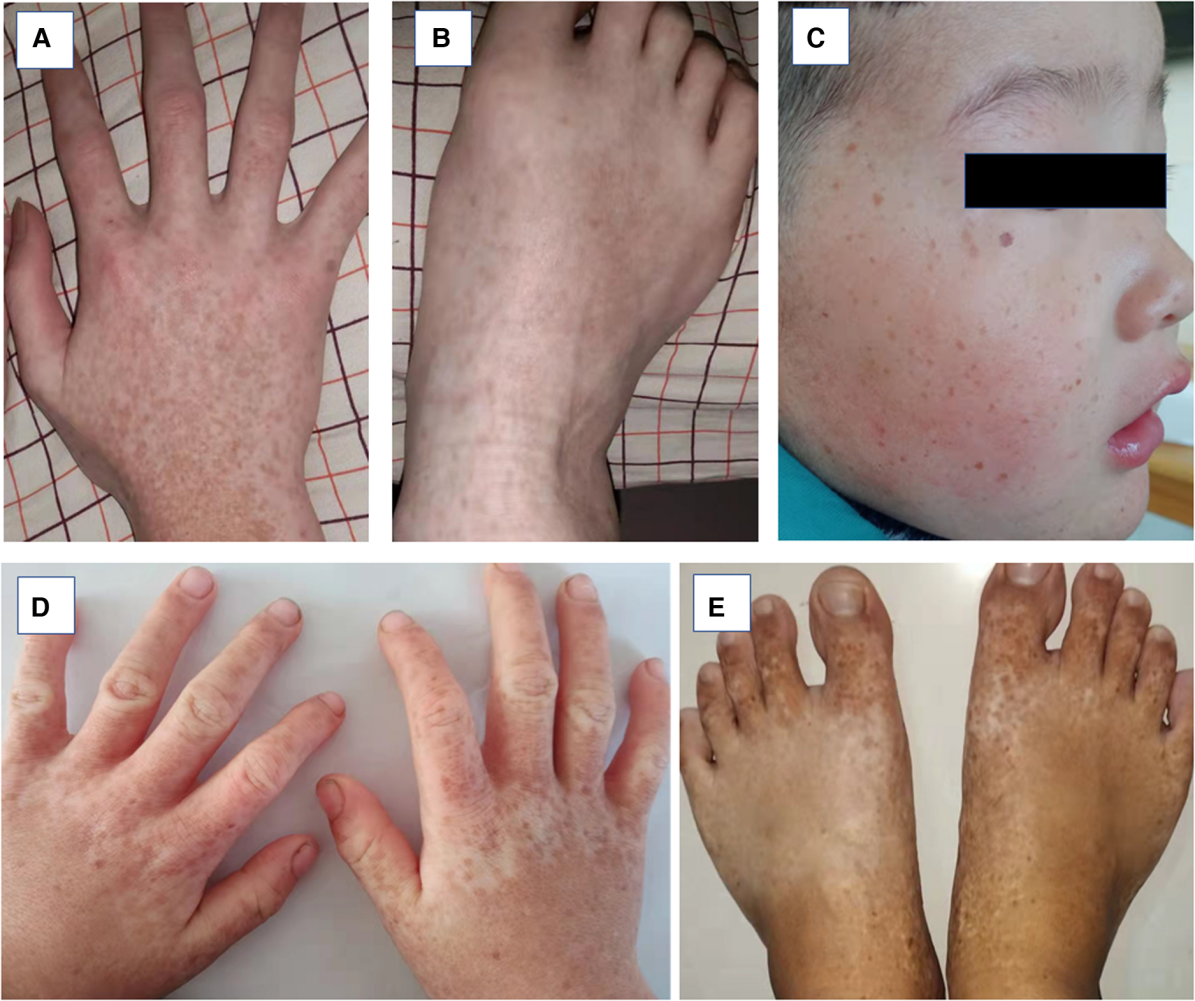
Clinical pheotypes of partly affected individuals. (**A** and **B**) Family 2-III:1 presented with back of hand and feet. (**C**–**D**) Family 3-II:1 presented back of hands and whole face. (**E**) Family 4-II:1 presented back of feet.

Patients with DSH of Han Chinese ethnicity were enrolled by medical specialists at the First Affiliated Hospital of Zhengzhou University (January 2018–December 2020). Of these, five probands showed that members of their pedigree had clinical manifestations of DSH. Among the five families, the fifth were sporadic cases in which the pedigrees did not reveal any history of DSH features in the previous three generations, whereas the remaining families had 1–5 members affected by DSH (Figure 1). In addition, 100 healthy and unaffected individuals were randomly selected for this study. All affected individuals had typical hyperpigmented and hypopigmented macules of irregular shapes and varied sizes on the dorsal aspects of their hands and feet. Family 1 proband, a 21-year-old boy, developed intermingled lesion of hyperpigmented and hypopigmented macules on the dorsal aspects of his hands and feet at the age of eight. Family 2 proband, a 10-year-old boy, manifested freckle-like macules with an irregular distribution of pigment spots on the dorsal aspects of his hands and feet and weak freckle-like macule on the face since the age of three. Family 3 proband, a 3-year-old boy with early onset, was diagnosed as hyper-pigmented and macules on dorsal of the extremities and freckle-like macule on the face by experienced dermatologists. The proband of family 4, a 12-year-old adolescent, displayed an irregular distribution of pigment spots on her hands and feet at the age of three. The proband of family 5, a 35-year-old male, developed similar phenotype as family 1 proband with hyperpigmented and macules on dorsal of the extremities. In addition, family 3 proband had hyperpigmentation spots on the arms. No other symptoms or signs were observed in any of the 14 patients. Detailed routine examinations of the patients in this study showed the progressive appearance of pigmented and depigmented macules (Figure 2). Prior to clinical evaluation and collection of patient images and data, written informed consent was obtained. Table 1 summarizes the clinical characteristics of the proband involved in this study.

**Table 1:** Clinical and molecular findings in this study.

No.	Incidence	AI	UI	Nucleotide and Amino acid substitution	Mutation location	Part of lesions	Mutation type	Protein domain	Predicted mutation effects		
									SIFT	Poly-phen 2.0	Mutationtaster
1	Familial	2	1	c.511G>T(p.E171*)	Exon2	extremities	Nonsense	z-alpha	NA	NA	D(1.0)
2	Familial	5	2	c.3385T>G(p.C1129G)	Exon14	extremities and face	Missense	ADEAMc	D(0.02)	D(0.999)	D(1.0)
3	Familial	3	1	c.3384G>C(p.W1128C)	Exon14	extremities and face	Missense	ADEAMc	D(0.001)	D(1.0)	D(1.0)
4	Familial	3	1	c.716G>A(p.R239Q)	Exon2	extremities	Missense	DSRM	D(0.004)	D(1.0)	D(1.0)
5	Sporadic	1	2	c.2080-1G>T	Intron 6	extremities	Splice-site	NA­	NA­	NA­	NA­

AI, affected individulas; UI, unaffected individuals; D, damaging; NA, no data available.

### 2.3. Samples and DNA extraction

Genomic DNA was extracted from EDTA-treated peripheral blood samples using the Mag-Bind® Blood & Tissue DNA HDQ 96 kit (Omega Bio-tek, GA, USA) according to the manufacturer’s protocol and quantified using an ultraviolet spectrophotometer Du800 (Beckman Coulter, Inc.).

### 2.4. Sequencing and mutational analysis

The present study examined five individual Chinese patients with DSH, showing an autosomal dominant pattern of inheritance. Genomic DNA was extracted from each patient and enriched using a customized panel. It was designed to capture 266 known genes related to dermatitis to investigate the genetic mutations present in DSH families. These genes were sequenced on the Illumina NextSeq500 system (Supplementary Table S1). The quality control of next-generation sequencing has been illustrated in Supplementary Tables S2, S3.

Burrows-Wheeler Aligner (BWA, version 0.7.5) was used to analyze raw data and align with the human hg19 reference sequence (18, 19). Polymorphisms including single nucleotide polymorphisms (SNPs) and indels were identified. ANNOVAR, a widely used tool for annotating sequence variants, was used to filter out subsets of SNPs reported in major human genomic variation databases (gnomAD/ExAC, dbSNP, 1,000 Genomes Project) and inherited disease mutation databases (HGMD and Clinvar) (20–22). Coding sequence alterations (exonic) and part of noncoding sequence variants (exon-intron boundaries) that present an unknown frequency or minor allele frequency <1% in these databases were reserved. SIFT (https://sift.bii.a-star.edu.sg/) (23), PolyPhen-2 (http://genetics.bwh.harvard.edu/pph2/) (24) and MutationTaster (http://www.mutationtaster.org/) (25) were used to predict the potential effects of novel missense/nonsense/frameshift variants. SWISS-MODEL (http://swissmodel.expasy.org/) was used to predict ADAR1 protein structure (26, 27). ClusterX (https://genome.ucsc.edu/) was used to evaluate the nucleotide conservation between species (28). Sequence variant interpretation was performed according to the ACMG/AMP recommendations for the standards and guidelines for sequence variant interpretation.

### 2.5. Polymerase chain reaction (PCR) amplification and Sanger sequencing

Coding exon regions of *ADAR1* were amplified using primers designed by Primer 5.0 software (Premier Biosoft, Palo Alto, CA, USA) and synthesized by Shanghai Shangon Co., Ltd. (Shanghai, China) (Table 2). The dGTP BigDye® Terminator sequencing kit (ABI, USA) and capillary electrophoresis instrument (ABI 3130XL, USA) were used for Sanger sequencing. One hundred unrelated healthy controls were sequenced for mutations to exclude the possibility of polymorphism in *ADAR1*. Presence of mutations were confirmed by identifying and contrasting against the sequences of the cDNA database. The novel mutations included three missense mutations (NM_001111.5: c.3385T>G, NM_001111.5: c.3384G>C, and NM_001025107.3: c.716G>A), one nonsense mutation (NM_001111.5: c.511G>T), and one splice-site mutation (NM_001111.5: c.2080-1G>T).

**Table 2:** Primers for validation.

Exon	Primers (5′-3′)	Product size
Exon 2	F: CAAGTGGACATCAGGGGTGT	497
	R: GCCTGAGCTGAGACTGCAA	
Exon 5−6	F:GGTCAGGCTCCTCAGTCAAA	599
	R:AGCACACCCTTGTTTTCCCT	
Exon 14	F:TGACCCCACACTTCCTCTCT	491
	R:GTAGATCCCTGCGGTAACGG	

## 3. Results

Screening of skin disease-related genes was performed using gene panel sequencing. In this study, we identified five new mutations of *ADAR1* among five Chinese families with DSH by NGS of the entire coding and flanking intronic sequences of the transcripts NM_001111.5 and NM_001025107.3. Direct sequence analysis of PCR products was performed to confirm the mutations. As shown in Table 1, the spectrum of mutations included three missense mutations (p.R239Q, pC1129G, and p.W1128C), one nonsense mutation (p.E171*), and one splice-site mutation (c.2080-1G>T), all of which were heterozygous. Upon searching the human gene mutation database (HGMD) and Ensemble database, we did not find a description related to these variants. All the variants segregated the affected individuals from 100 healthy individuals, confirming that they were not polymorphisms.

**Figure 3:**
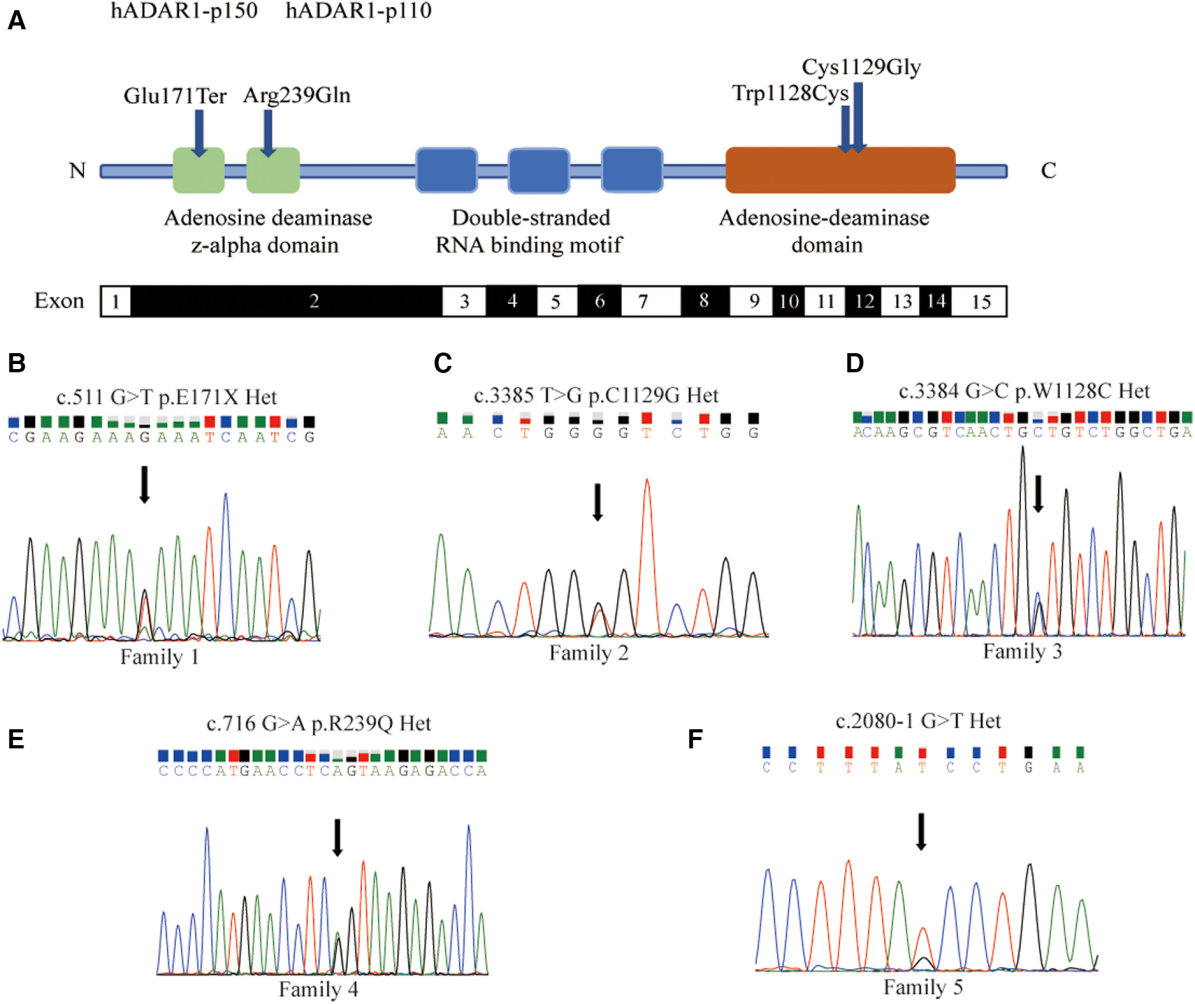
Five new mutations of *ADAR1* gene and direct sequencing for proband. (**A**) Schematic representation of the structure of *ADAR1* gene mutations. The functional domains (Z-alpha, DRBMs, ADEAMc) are indicated in the figure by different colours. The identified mutations in this Study are indicated by dark blue symbols. (**B**) The 511 nucleotide of the transcript NM_001111.5 was mutated from G to T, and the corresponding amino acid was mutated from glutamate to the stop codon. (**C**) The 3,385 nucleotide was mutated from T to G, and amino acid was mutated from cysteine to glycine. (**D**) The 3,384 nucleotide was mutated from G to C, and amino acid was mutated from tryptophan to cysteine. (**E**) The 716 nucleotide of NM_001025107.3 was mutated from G to A, and amino acid was mutated from arginine to glutamine. Missense mutation at nucleotide 716 of Exon2 resulted in the 239th amino acid mutation of from arginine to glutamine. (**F**) The 2080-1 nucleotide was mutated from G to T.

**Figure 4:**
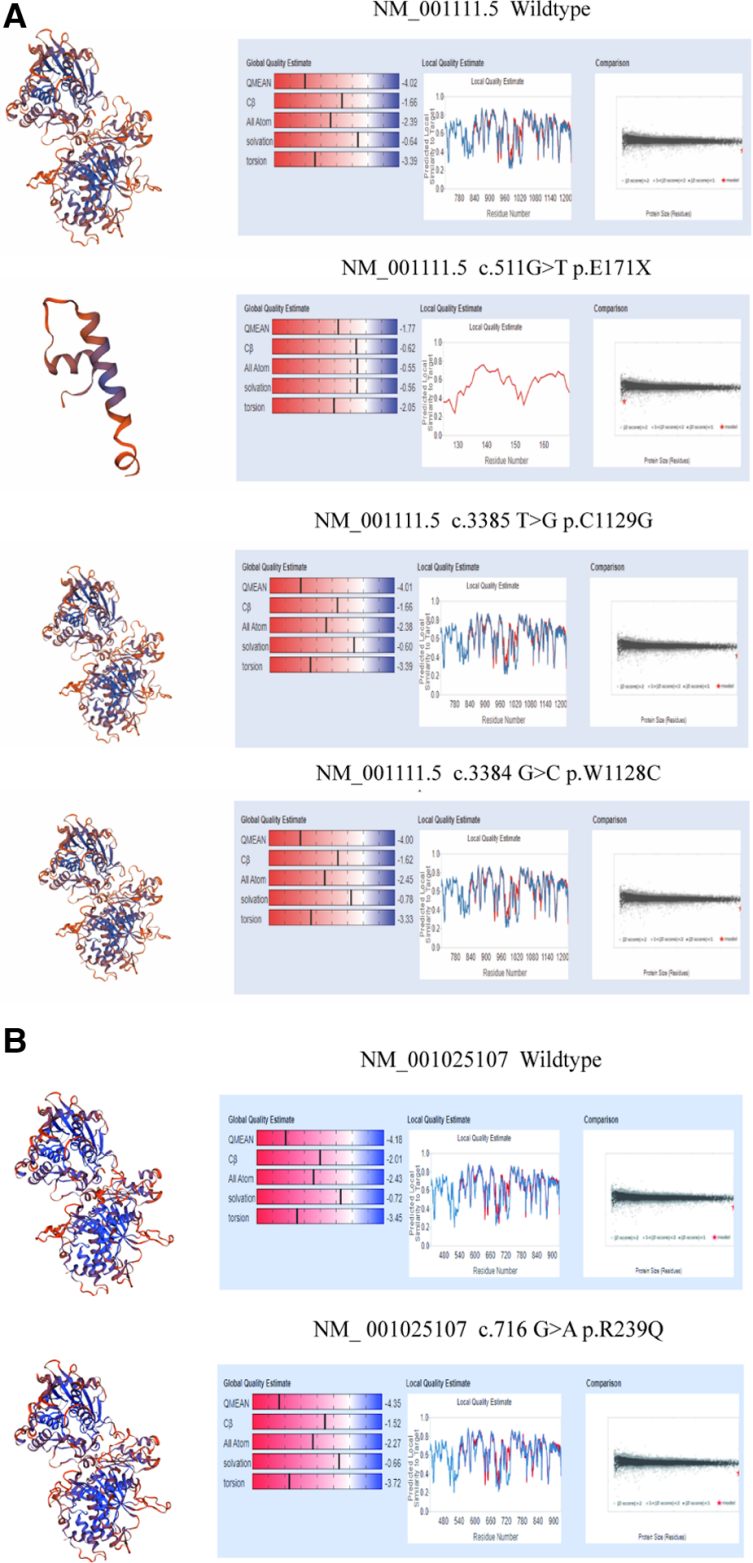
Structure modeling of wild-type and mutation of *ADAR1* gene with SWISS-MODEL. (**A**) 3D model of wild type ADAR1 protein (NM_001111.5 transcript, NP_001102.3) (**B**) 3D model of variant ADAR1 protein (NM_001111.5 transcript, NP_001102.3) c.511G>T(p.E171X) of family1. (**C**) Protein structure of ADAR1 protein (NM_001111.5 transcript, NP_001102.3) c.3385T>G(p.C1129G) of family2. (**D**) 3D model of variant ADAR1 protein (NM_001111.5 transcript, NP_001102.3) c.3384G>C(p.W1128C) of family3. (**E**) Protein structure of wild type ADAR1 protein (NM_001025107.3 transcript, NP_001020278.1). (**F**) 3D model of variant ADAR1 protein (NM_001025107.3 transcript, NP_001020278.1) c.716G>A(p.R239Q) of family4.

**Figure 5:**
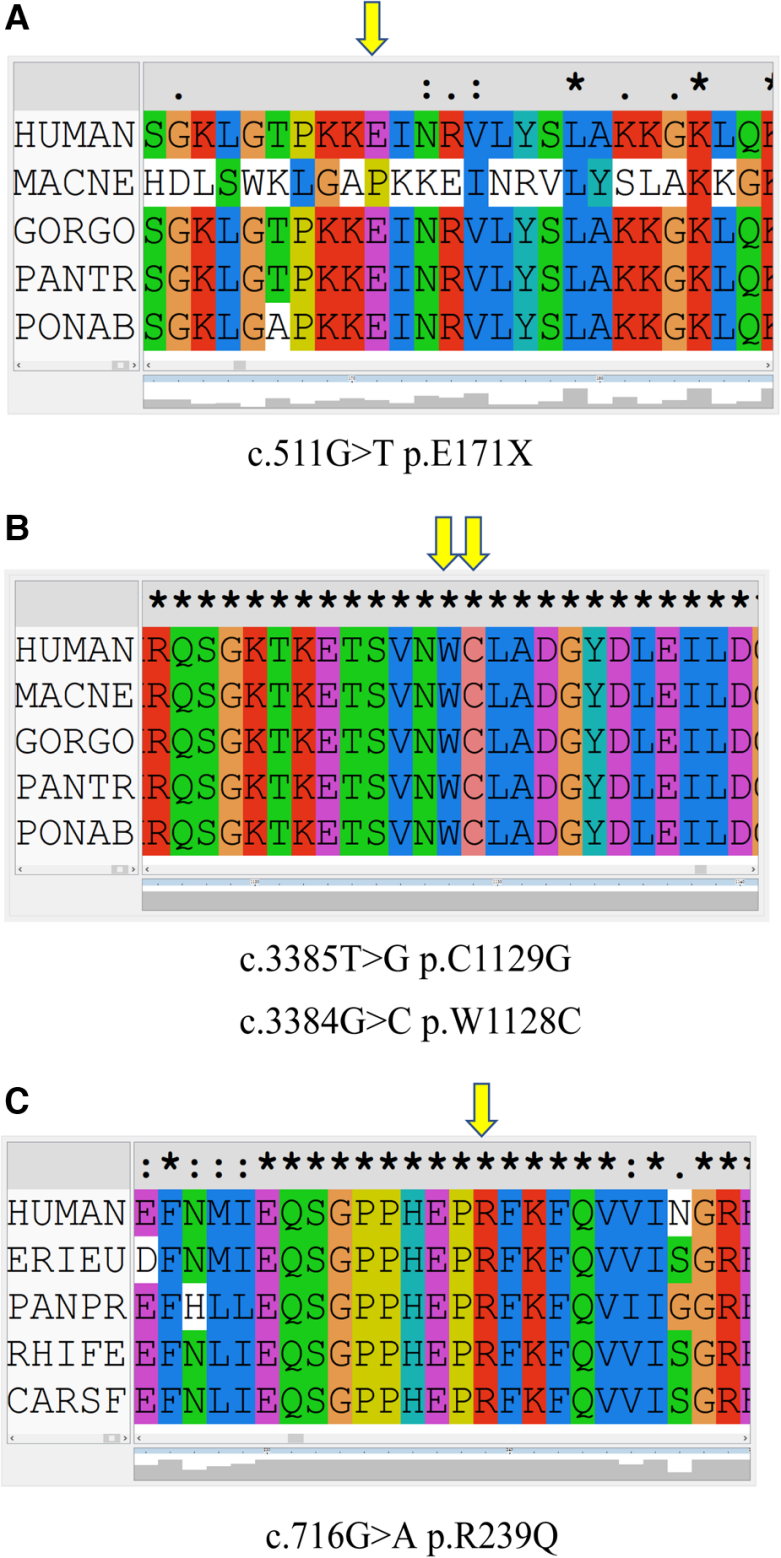
Conservation of amino acid sequences in the corresponding mutation of *ADAR1* between species. The yellow arrow represents the amino acid at the mutated site. (**A**) c.511G>T (p.E171*) mutation in *ADAR1*. (**B**) c.3385T>G (p.C1129G) and c.3384G>C (p.W1128C) mutations in *ADAR1*. (**C**) c.716G>A (p.R239Q) mutation in *ADAR1*.

In family 1, one nonsense mutation, c.511G>T (p.E171*), was detected in exon 2 of *ADAR1* (Figure 3B). This mutation was predicted to result in a truncated ADAR1 protein lacking 956 amino acid residues. This mutation leads to the termination of the translation of ADAR1 protein in the Z-alpha and double-stranded RNA-binding motif (DSRM) domains, which will markedly affect the structure and function of the protein (Figure 4B). According to the ACMG guidelines and criteria,the variant was judged as a suspected pathogenic variant, PM1 + PM2 + PP3. In families 2 and 3, two missense mutations, c.3385T>G and c.3384G>C, leading to cysteine at position 1,128 and glycine at position 1,129 were found within exon 14; these were detected in related family members and not in healthy individuals (Figures 3C,D). Both mutations occurred in the NM_001111.5 transcript. SWISS-MODEL modeling revealed that the protein structure did not change significantly after these mutations (Figures 4C,D). According to the ACMG guidelines and criteria, both variants were determined to suspected pathogenic variants, PM1 + PM2 + PP3. Moreover, NGS sequencing of *ADAR1* revealed a missense mutation, c.716G>A, at the end of exon 2 in family 4, and this mutation was confirmed by Sanger sequencing (Figure 3E). Missense mutation of nucleotide 716 of exon 2 resulted in change of amino acid 239 of ADAR1 to glutamine, and the DSRM domain of ADAR1 protein was also altered. The mutation occurred in the NM_001025107.3 transcript. According to the ACMG guidelines and criteria, the variants was predicted to suspected pathogenic variants, PS1 + PM1 + PM2 + PP3. Moreover, SWISS-MODEL predicted that the structure of this protein would not change significantly by this mutation. Compared with homologous proteins of human and primates, the four novel exonic mutations identified in our study occurred in highly evolutionarily conserved residues within the functional domain of ADAR1 (Figure 5). Moreover, one novel splice-site mutation in the intron region, c.2080-1G>T, was found to be pathogenic (Figure 3F). The prediction of the ACMG guidelines and criteria was suspected pathogenic variants, PVS1 + PM2 + PP4.All mutations were predicted to be damaging. The pathogenicity of the variants was predicted using the following tools: SIFT, PolyPhen 2, and MutationTaster (Table 1).

## 4. Discussion

DSH is an autosomal dominant skin disorder characterized primarily by hyperpigmentation and hypopigmentation spots on the dorsal extremities (29). It may also be associated with other complications, including dystonia, acromegaly, psoriasis, and depression (30). DSH is characterized by a symmetrical distribution of pigmentation and pigmented spots on the dorsal sides of the hands and feet. This pigmentation occurs in infancy or early childhood and usually stops progressing before puberty. Histologically, a focal increase or decrease in the amount of melanin or the number of melanocytes in the basal layer of the skin is observed. In our study, all the probands developed freckle-like macules on the face with an irregular distribution of pigment spots on the dorsal aspects of his hands and feet. In addition, family 2 is part of a three-generation pedigree that includes five affected individuals (Figure 1B). Mutations in *ADAR1* comprise the genetic basis of DSH. However, the mechanism through which *ADAR1* mutations cause DSH is still unclear.

NGS has revolutionized the era of sequencing. The various advantages of the method can be mentioned as higher sensitivity to detect frequency variants, faster turnaround time for high sample volumes, comprehensive genomic coverage, higher capacity with sample multiplexing, ability to sequence hundreds to thousands of genes or gene regions simultaneously. However, as a high-throughput sequencing method it is confronted with numerous disadvantages which are expected to solve such as large storage capabilities, sophisticated bioinformatics systems, high speed connections to internet and fast data processing infrastructures, each of which can be costly. Despite the challenges and questions, the application and data provided by NGS are vital to make it valuable which are determination of gene expression levels, novel gene and transcripts, finding gene exons. Thus, NGS is still an excellent method for detecting genetic variants and has much more potential in the research space than it does in the clinical space, especially for rare disease testing (31). In our study, we used NGS method to identify the mutations of 266 gene panel instead of only targeting the ADAR1 gene. It was expected to explore more genetics variant loci, which may be more meaningful for elucidating the functional mechanism of DSH.

*ADAR1* gene, located at 1q21.1–q21.2, encodes an RNA-specific adenosine deaminase that represents a type of RNA-editing enzyme; it spans 30 kb, contains 15 exons, and the encoded protein contains 1,226 amino acid residues (Figure 3A) (32). Two adenosine deaminase domains, Zα, and three DSRMs that are spread over exon 2 and exons 2–7 are responsible for RNA editing by site-specific deamination of adenosine (33). The tRNA-specific double-stranded RNA adenosine deaminase (ADEAMc) domain is encoded in exons 9–14. ADAR catalyzes the deamination of adenosine in double-stranded RNA to form inosine (A-to-I editing), which is a type of post-transcriptional modification widely present in mammals (34). Every DBRM domain can enhance the efficiency of editing, and the ADEAMc domain is essential for editing (35). Since inosine is recognized as guanosine in the process of translation, the editing of the A-to-I RNA-coding sequence may lead to a change in amino acids and protein function. Thus, A-to-I editing may change the splicing patterns, RNA structure, and stability (36). Abnormal RNA editing may cause the differentiation of melanoblasts into hypoactive melanocytes, thus establishing an irregular colonization of the lesioned skin (37).

To date, a total of 267 clinically significant sequence variants of *ADAR1* have been obtained from HGMD linked to dbSNP, consisting of 147 missense and nonsense mutations, 27 splicing mutations, 2 regulatory mutation, 61 small deletions, 25 small insertions, 4 small indels, and 1 gross insertion (5, 11, 12, 38–49). Analysis based on the Clinvar, UniprotKB, HGMD, and DBSNP databases showed that the five novel *ADAR1* mutations identified in this study had not been reported previously. To date, over half of the known missense mutations are located within the ADEAMc domain encompassing amino acids 839–1,222 (50). The two missense mutations identified in our study were also present in this domain. These findings suggest that the ADEAMc domain is critical for the function of this enzyme and may be defined as a potential mutational hotspot region within *ADAR1* (48, 50, 51). To date, 32 nonsense mutations have been reported in *ADAR1*, and they seem to be randomly spread throughout the genome having apparent unifying connections.

SWISS-MODEL analysis was used to evaluate homology modeling of the tertiary structure of wild-type and mutant ADAR1 and examine the impact of gene mutations on the tertiary structure of the protein (27). Our SWISS-MODEL modeling showed that the nonsense mutations in exon 2 resulted in significant truncation of the protein, leading to complete deletion of the DSRM and ADEAMC domains of the ADAR1 protein that are considered key structural domains for its function. The other three mutations (p.R239Q, p.W1128C, and p.C1129G) resulted in structural alterations of DSRM and ADEAMC domains of ADAR1 protein without significant changes in spatial structure; however, the prediction tools, SIFT, Polyphen-2, and MutationTaster, showed that all of them were pathogenic mutations (Table 1).

To date, cumulative studies have shown that mutations in *ADAR1* contribute to several hereditary diseases, including DSH, AGS, bilateral striatal necrosis, dystonia, athetosis, Leigh-like syndrome, and others, whose hereditary pattern may change (52, 53). Table 3 summarizes the distribution of mutations in *ADAR1* according to HGMD. DSH is characterized by a symmetrical distribution of pigmentation and pigmented spots on the dorsal sides of the hands and feet. This pigmentation occurs in infancy or early childhood and usually stops progressing before puberty. Histologically, it is characterized by a focal increase or decrease in the amount of melanin or the number of melanocytes in the basal layer of the skin. The data from HGMD showed that the majority of known mutations were related to DSH. For decades, most people have attributed DSH to haploinsufficiency and dominant-negative effects of mutant *ADAR1*. In addition, AGS, an autosomal recessive autoimmune disease that affects the nervous system (with complications such as intracranial calcification, leukystrophy, and severe developmental delay), is caused by *ADAR1* mutation (12). Kono et al. (54) reported a case of a Japanese patient with AGS and phenotypic characteristics of DSH (neurological symptoms and brain calcification), who was compound heterozygous for *ADAR1* mutation, suggesting that homozygous *ADAR1* or compound heterozygous *ADAR1* mutations may lead to a combination of AGS and DSH in East Asian patients, but only in non-East Asian patients.

**Table 3:** The profiles of ADAR1 mutation in HGMD.

Mutation type	Number of mutations
Missense/nonsense	111
Splicing	17
Regulatory	1
Small deletions	50
Small insertions	21
Small indels	2
Gross deletions	0
Gross insertions/duplication	1
Public total (HGMD Profeesional 2021.2) total	267
Disease/phenotype	Number of mutations
Dyschromatosis symmetrica hereditaria	182
Aicardi-Goutières syndrome	15
Bilateral striatal necrosis	2
Dyschromatosis symmetrica hereditaria?	1
Dystonia, athetosis and Leigh-like syndrome	1
HBsAg seroclearance, association with	1
IFN gamma response to Rubella, association with	1

Depending upon the expression patterns, two isoforms of ADAR1, an interferon-inducible, cytoplasmic protein with a molecular mass of 150 kDa (p150) and a constitutively expressed nuclear protein with a molecular mass of 110 kDa (p110) are synthesized by translation initiation at alternative methionine-encoding AUG codons in mammalian cells (3, 55). The p150 isoform that contains a nuclear export signal at the N-terminal of the Zα domain is the full-length protein predominant in the cytoplasm (56). The p110 isoform consists of three DRBMs and a catalytic domain and is mainly localized in the nucleus owing to the presence of a nuclear localization signal (57). So far, five mutations of *ADAR1*, including p.E171* identified in our study, have been reported at the 5′ side of codon 296 (12, 58–60). However, the distinct roles of the p150 and p110 isoforms are still obscure, and the specific roles of these two isoforms in the pathogenesis of DSH are poorly understood. Recently, Zhang et al. (11) reported that a novel frameshift mutation (p.R91fsX123) could eliminate the expression of the p150 isoform through nonsense-mediated mRNA decay, although it did not change the expression of p110 isoform; this indicates that the ADAR1 p150 isoform might be a determinant of DSH.

The chromatic aberration in DSH is mainly confined to areas exposed to sunlight, which affects the appearance of the skin. Current treatments have not been successful in alleviating this problem. An increasing number of studies have shown that topical application of sunscreen can be successful in controlling hereditary color symmetry disorder (61). Currently, there is no complete cure for DSH, and treatment is limited to supportive care, presenting many challenges in clinical practice (62). Therefore, the establishment of a reliable diagnosis of DSH is crucial for the identification of DSH subtypes and development of therapies. In the future, It is possible to evaluate the genetic status of an embryo, produced by *in vitro* fertilization before the embryo is transferred into the woman’s uterus. Preimplantation genetic testing for aneuplodiy (PGT-A) and for monogentic/single-gene disease (PGT-M) could provides information about the embryo’s chromosomes and seaching for the presence of a specific, disease-causing gene (63–65). Thus, the intervention in the embryonic period is an effective prevention strategy. Furthermore, parenteral genetics screening/diagnosis could be the best methods for managing this disease, which currently has no therapy. The screening result associated with the gene would help us identify genotype-phenotype correlations and lead to clinical trials in the future. Our study may contribute to the timely and improved clinical management of patients with DSH.

## 5. Conclusion

In brief, our study indicates the importance of genetic testing for DSH diagnosis and enriches the spectrum of known variants regarding the genetic background of this disease. The present study opens a new era for understanding DSH genotype/phenotype correlations as well as genetic counselling for this disease.

## Data Availability

The raw data supporting the conclusions of this article will be made available by the authors, without undue reservation.
