# Supplementary material for: Identification of five novel variants of ADAR1 in dyschromatosis symmetrica hereditaria by next-generation sequencing
Source: Front Pediatr. 2023 Jul 5;11:1161502. doi: 10.3389/fped.2023.1161502 (PMC10354868; doi:10.3389/fped.2023.1161502)
Supplement: Supplementary file 1 [file Table1.docx]

| AAGAB | CLCF1 | ERCC1 | IL36RN | MAP2K1 | PTCH1 | STK4 |
| --- | --- | --- | --- | --- | --- | --- |
| ABCA12 | CLDN1 | ERCC2 | ING1 | MAP2K2 | PTEN | STS |
| ADAM17 | CNTN2 | ERCC3 | INSR | MBTPS2 | PTPN11 | TBXA2R |
| ADAMTS2 | COL14A1 | ERCC4 | IRF2 | MC1R | PTPN6 | TEK |
| ALAS2 | COL17A1 | ERCC5 | ITGA3 | MITF | PVRL1 | TERT |
| ALDH18A1 | COL1A1 | ERCC6 | ITGA6 | MLPH | PVRL4 | TGFBI |
| ALDH3A2 | COL1A2 | EWSR1 | ITGB4 | MSMO1 | PYCR1 | TGM1 |
| ALOX12B | COL3A1 | EXPH5 | ITPKC | MSX1 | RAB27A | TGM5 |
| ALOXE3 | COL5A1 | FBLN5 | JUP | MVK | RECQL4 | TINF2 |
| ANTXR2 | COL5A2 | FBN1 | KAL1 | MYH8 | RIN2 | TLR1 |
| AP3B1 | COL6A5 | FECH | KCTD1 | MYO5A | RNASEH2A | TLR5 |
| APOE | COL7A1 | FERMT1 | KIT | NFKBIA | RNASEH2B | TMC6 |
| AQP5 | COX7B | FGFR3 | KLK7 | NHP2 | RNASEH2C | TMC8 |
| ARHGAP31 | CREB1 | FH | KRT1 | NIPAL4 | RNF114 | TNFRSF10B |
| ARHGAP6 | CSTA | FLG | KRT10 | NIPBL | RSPO1 | TNXB |
| ARPC3 | CTNNB1 | FUS | KRT13 | NLGN4X | RTEL1 | TP53 |
| ASIP | CTSC | GJA1 | KRT14 | NLRP1 | SAMHD1 | TP63 |
| ASPRV1 | CYLD | GJB2 | KRT17 | NOD2 | SART3 | TRAF6 |
| ATF1 | CYP26C1 | GJB3 | KRT2 | NOP10 | SAT1 | TREX1 |
| ATP2A2 | CYP4F22 | GJB4 | KRT4 | OCA2 | SHOC2 | TSC1 |
| ATP2C1 | DDB2 | GJB6 | KRT5 | OSMR | SLC11A1 | TSC2 |
| ATP6V0A2 | DKC1 | GPR143 | KRT6A | PAX3 | SLC24A5 | TUBG1 |
| AXIN2 | DOCK9 | HOXA2 | KRT6C | PDCD1 | SLC26A4 | TYR |
| B4GALT7 | DSC3 | HOXC13 | KRT81 | PDE11A | SLC27A4 | TYRP1 |
| BLOC1S3 | DSG1 | HPS1 | KRT83 | PDGFRA | SLC29A3 | UROD |
| BMS1 | DSG4 | HPS3 | KRT85 | PHF11 | SLC39A13 | UROS |
| BRAF | DSP | HPS4 | KRT86 | PIGL | SLC39A4 | USB1 |
| C10orf11 | DST | HPS5 | KRT9 | PIK3CA | SLC45A2 | VPS33B |
| C12orf10 | DTNBP1 | HPS6 | LAMA3 | PKP1 | SLC4A11 | VSX1 |
| CARD14 | EDA | HR | LAMB3 | PLEC | SLURP1 | WDR35 |
| CD22 | EDA2R | HRAS | LAMC2 | PLOD1 | SMC1A | WNT10A |
| CDH3 | EDAR | IFT122 | LIPH | PMS2 | SMC3 | WRAP53 |
| CDSN | EDARADD | IFT43 | LIPN | PNPLA1 | SOS1 | XPA |
| CERS3 | EDNRB | IKBKG | LMNA | POLH | SOX18 | XPC |
| CHRNA1 | EFEMP2 | IL17RA | LMX1B | PORCN | SPINK5 | XYLT2 |
| CHRND | ELN | IL1RN | LOR | PRKAR1A | SSH1 | ZEB1 |
| CHRNG | ELOVL4 | IL20RA | LPAR6 | PRKDC | ST14 | ZMPSTE24 |
| CHST8 | EMILIN2 | IL31RA | LTA | PSTPIP1 | STK11 | ZNF750 |
| ADAR1 |  |  |  |  |  |  |

Supporting information:

Table S1. Gene list of the NGS penal
